# Interspecific Aggressive Behaviour of Invasive Pumpkinseed *Lepomis gibbosus* in Iberian Fresh Waters

**DOI:** 10.1371/journal.pone.0088038

**Published:** 2014-02-05

**Authors:** David Almeida, Raquel Merino-Aguirre, Lorenzo Vilizzi, Gordon H. Copp

**Affiliations:** 1 Salmon and Freshwater Team, Cefas, Lowestoft, United Kingdom; 2 Centre for Conservation Ecology and Environmental Change, Bournemouth University, Poole, United Kingdom; 3 Department of Ecology, Complutense University of Madrid, Madrid, Spain; 4 Ichth-Oz Environmental Science Research, Irymple, Victoria, Australia; 5 Environmental and Life Sciences Graduate Program, Trent University, Peterborough, Ontario, Canada; CNRS, Université de Bourgogne, France

## Abstract

Pumpkinseed *Lepomis gibbosus* (L.) are successful invaders in Europe, where this species exerts multiple ecological effects, mainly through trophic interactions. Behavioural interference represents a potential impact for native fauna and this is of particular conservation concern in the Iberian Peninsula because of the highly valuable endemic fauna inhabiting streams of this region. However, aggressive interactions have not previously been examined under natural conditions in Iberian fresh waters. To address this gap in knowledge, the aim of the present study was to assess the effect of pumpkinseed aggression on endemic fauna of an Iberian stream, the River Bullaque (central Spain). In September 2009, we analysed the aggression and environmental contexts of these behavioural interactions by snorkelling: aggressor size, aggression type, shoal size, previous activity to aggression, recipient species, response to aggression, microhabitat structure and prey availability. Small pumpkinseed displayed more threat and fewer pursuit behaviours relative to medium and large individuals, reflecting an ontogenetic behavioural shift from low to high aggression intensity. Small aggressors came from large shoals, with bottom feeding being the most frequently observed activity prior to an aggressive interaction; whereas large pumpkinseed were less gregarious and they were mostly ambulating within the water column prior to aggression. Recipient species of aggression included non-native crayfish and fishes, and more importantly, endemic fishes and frogs. Retreat was the most common response to aggression, irrespective of aggressor size. Small pumpkinseed displayed aggressive behaviours over coarse substrata containing elevated macrobenthos biomass; whereas aggression by large individuals was observed in deeper waters. These findings suggest that small and large pumpkinseed exert a high impact on other stream residents through aggression in competition for food and territory defence, respectively. This study highlights the usefulness of direct observations in the wild for assessing the effects of behavioural interference of invasive fishes on Iberian aquatic communities.

## Introduction

One of the most widely-established introduced fishes in European fresh waters is the pumpkinseed *Lepomis gibbosus* (L.) [1], a North American centrarchid that possesses virtually all of the attributes associated with successful invaders [2], [3], including great plasticity of biological traits and behaviour [4], [5]. This invasion success is apparent in the species’ broad introduced range in Europe, which extends from southern Norway to the Mediterranean region [1], [6], and in the wide variety of invaded ecosystems (i.e. ponds, lakes, reservoirs and all sizes of water courses). Until recently, the majority of research on the species’ environmental biology has addressed still-water populations, but studies that include or focus on non-native stream-dwelling pumpkinseed are on the increase [7]–[12].

Assessments of this species’ potential impacts are mainly related to dietary overlap with native species (e.g. [13]–[16]), including studies of some stream systems [9], [17]. Also, telemetry-based research represents a useful individual-based technique to assess interactions between introduced pumpkinseed and native species in European streams [9], [12], [18]. Thus, a study of pumpkinseed in a small tributary of the River Ouse (Sussex, England) suggested that, where this invasive species was in high density, there were differences in stream food-web structure, proportional representation of fish traits and riparian spider community composition [9]. However, these differences could not be attributed solely to pumpkinseed presence, and interactions with native brown trout *Salmo trutta* L. suggested that the two species co-habit the stream without inter-specific association due to their use of different parts of stream pools [12]. Recent attempts to evaluate other impacts of pumpkinseed introductions in European ecosystems include a study of an Iberian wetland, where the establishment of pumpkinseed was linked to a reduction in the size and number of zooplankton, leading to increased turbidity, chlorophyll *a*, total phosphorous and nitrogen relative to sites without pumpkinseed [19].

Apart from these ecological effects, behavioural interference represents a potential adverse impact of non-native fishes on native species [20]. Aggression is the main mechanism of this interaction, leading to disruption of the natural behaviour of native fauna during feeding, breeding or searching for refuge [21], [22]. This is of particular conservation concern in the Iberian Peninsula because of the highly valuable endemic fauna inhabiting streams of this region [23], [24]. All known previous studies of aggressive interactions between non-native fishes and Iberian species have been carried out in the laboratory or in mesocosm facilities (e.g. [25], [26]), which do not encompass the range of ecological factors present in the natural environment [27]. Whereas, *in situ* underwater observations are implemented under natural environmental conditions and the results are therefore more realistic. In relation to this, snorkelling is a promising technique for assessing the impact of non-native fishes, as this is a method with a low level of disturbance on fish behaviour [21].

To address the gap in knowledge on aggression impacts under natural conditions and to provide insights on the autoecology of this invasive fish, the aim of the present study was to assess the effect of pumpkinseed aggression on endemic fauna of an Iberian stream. Specifically, we carried out snorkelling surveys to analyse the aggression and the environmental contexts of these behavioural interactions: aggressor size, aggression type, shoal size, previous activity to aggression, recipient species, response to aggression, microhabitat structure and prey availability.

## Materials and Methods

### Ethics Statement

No permits were required for the field sampling, as surveys did not include collection or manipulation of protected species and complied with all relevant regulations of Europe and Spain. The study area was public and not protected. Fish observations and invertebrate collection were performed by trained personnel (D. Almeida, R. Merino-Aguirre), who had already carried out previous surveys in the same water course (e.g. [5], [28]). Thus, field procedures did not cause any adverse effects on the aquatic wildlife of the sampling sites.

### Study Area

The study was undertaken in the River Bullaque, a first-order tributary (94 km length, 1019 km^2^ catchment area, altitude: 550–620 m a.s.l.) of the River Guadiana (central Spain), which drains a region characterised by Precambric and Palaeozoic slates and quartzites, and by a Mediterranean climate: rainfall is abundant from late autumn to spring (500–800 mm), whereas summer is hot and dry. Annual mean temperature ranges from 9 to 14°C. The lowest temperatures are registered in December (–5°C), whereas the highest value (43°C) occurs in August. However, most of the River Bullaque is not subjected to the wide seasonal variations in water level that are typical of small Mediterranean water courses because its discharge is regulated by the Torre Abraham Dam, which maintains a continuous and relatively invariable discharge regime throughout the year (see [28] for discharge profiles). The River Bullaque is typical of many regulated small rivers in the Mediterranean region of the Iberian Peninsula. The particular biotic and abiotic characteristics of the study area have been previously described in Almeida et al. [5], [8], [28].

The fish assemblages of the River Bullaque include several species of cyprinids and cobitids endemic to the Iberian Peninsula, such as the long-snouted barbel *Luciobarbus comizo* (Steindachner), Iberian small-head barbel *L. microcephalus* (Almaça), calandino *Squalius alburnoides* (Steindachner), southern Iberian chub *S. pyrenaicus* (Günther) and southern Iberian spined-loach *Cobitis paludica* (de Buen). Also present are endemic amphibians such as Perez’s frog *Pelophylax perezi* (López-Seoane) and non-native species such as eastern mosquitofish *Gambusia holbrooki* Girard, largemouth bass *Micropterus salmoides* (Lacépéde) and red-swamp crayfish *Procambarus clarkii* (Girard).

### Field Sampling

Field sampling was carried out in September 2009, a hydrologically average year for the study area [29], which increased the potential generality of the present findings. The first three weeks of September were selected because the pumpkinseed breeding season in the River Bullaque has ended [5], thus avoiding bias due to nest-guarding aggressive behaviours. Moreover, water temperature is still high in the end of summer, enhancing the likelihood of biotic interactions due to elevated native and non-native species activity [30]. Also, the high water temperature provides more comfortable conditions for the underwater observer (D. Almeida), permitting snorkelling surveys of longer duration.

We selected 18 sampling sites (500 m river length) along a 25 km river section downstream from Torre Abraham Dam, which consisted of different mesohabitats (i.e. run, riffle, pool) to include the existing environmental variability. Furthermore, their hydrological conditions (i.e. current, turbidity) during the sampling period allow underwater visibility to be highly suitable for snorkelling [21]. Direct underwater observations of pumpkinseed were made using a mask, snorkel and dry-suit, and moving in a downstream-to-upstream direction (adapted from standard procedures of [31]). Once an aggressive interaction (event) was observed for a particular pumpkinseed, the aggression and the environmental contexts were recorded.

Evaluation of aggression context included ‘Aggressor’ size, ‘Aggression’ type, ‘Shoal’ size, previous ‘Activity’, ‘Recipient’ species identity and ‘Response’ of the Recipient species. The size of the Aggressor was recorded in total length (TL) by size class (small: <70 mm; medium: 70–120 mm; large: >120 mm TL) consistent with the size distribution of the local pumpkinseed population [5], [8] and easily distinguishable by the underwater observer. The type of Aggression towards a Recipient species was adapted from Caiola and de Sostoa [26], Miller [32] and Hazelton and Grossman [33], and was recorded as follows (from least to most intensive): ‘Threat’ = slow approach, parallel swimming, fin erection and flaring; ‘Attack’ = rapid approach, charge with butting, pushing or even biting; and ‘Pursuit’ = aggressor pursues and tries to chase the recipient. The most intensive level of aggression was recorded in the event that the same aggressor displayed more than one type of aggressive behaviour. The size of the Shoal from which the Aggressor originated was classed as: <5, 5–10 and >10 individuals. The Activity of the Aggressor prior to the aggressive event was recorded as: bottom feeding (BF), water column feeding (WCF), bottom movement (BM) and water column movement (WCM). Recipient species identity included: red-swamp crayfish, *Luciobarbus* spp., calandino, southern Iberian chub, southern Iberian spined-loach, eastern mosquitofish, largemouth bass and Perez’s frog. Response of the Recipient species was also categorical: no response (NR), aggression (AGR), retreat with return (RR) and retreat with no return (RNR). Recipient species size was not recorded because of the high number of variables being measured by the observer during each aggressive event and also because low variability in recipient species total length was observed during previous snorkelling surveys [5].

The environmental context of each aggressive event included microhabitat structure and prey availability. Microhabitat structure encompassed: ‘Focal height’ of the Aggressor in the water column (distance to the bottom estimated as a percentage of depth, with 100% at the surface); water ‘Depth’ (cm); water ‘Velocity’ at the focal height (m s^–1^); ‘Distance’ of the Aggressor to the nearest bank (m); submerged ‘Vegetation’ cover (% of the area of a circle with a 0.6 m radius around the aggression point); and percentages of five substratum categories (as per [34]) determined visually in the same 0.6 m radius area, from which a ‘Coarseness’ index was calculated (adapted from [35]), ranging from 1 to 5, with 5 as the coarsest value. Prey availability was measured as biomass (dry mass, DM) of: ‘Zooplankton’ (mg DM m^–3^) and ‘Macrobenthos’ (g DM m^–2^); which were collected as per Almeida et al. [28] using zooplankton tubes and Surber samplers (40 and 250 µm mesh, respectively). Laboratory procedures regarding invertebrate processing were performed as per Almeida et al. [28]. Only the most important taxa of prey items for pumpkinseed were considered when estimating prey availability in the study area (see [8]).

For further information regarding data collection, the observer quickly noted the codes for the six parameters of the aggression context (see details above) and the focal height of every aggressive event on an underwater data board. For the remaining parameters of the environmental context, the observer indicated the direction to the aggression point by using his body line and noting the distance to that point (±30 cm). The observer passed the particular data sheet to a research assistant (R. Merino-Aguirre), who used a graduated telescopic pole (up to 4 m) to measure the distance to the aggression point and then waded in that direction. At that point, the assistant measured water velocity (Global Water, Flow Probe 101) and used the graduated telescopic pole to measure water depth and distance to the nearest bank, as well as to mark out the 0.6 m radius, within which submerged vegetation and substratum composition were assessed visually. Finally, the assistant collected samples of zooplankton and macrobenthos by netting (see details above). To avoid pseudo-replication, i.e. repeated observations of the same individual fish, the observer and the assistant moved upstream 5 m as quickly as possible after collecting data for a particular aggressive event. This methodology was adapted from previous studies on the assessment of fish microhabitat use in Iberian rivers by snorkelling (e.g. [36]) and applied by the observer in previous snorkelling surveys of pumpkinseed in this study area [5], [21]. Overall, the above procedures and observer experience allowed for accurate assessments of the aggression and the environmental contexts.

### Statistical Analyses

‘Sampling site’ was not accounted for as a factor within the analytical models because this effect was not significant, according to previous investigations in the study area and preliminary statistical analyses. Specifically, size structure of pumpkinseed and prey availability were assessed in Almeida et al. [8]; abundances and composition of fish and crayfish assemblages in Almeida et al. [37]; and microhabitat structure in Almeida et al. [28]. For these previous studies, the ‘site effect’ was not significant, as neither differences nor associations were found for the mentioned parameters (i.e. Aggressor size, Recipient species, microhabitat structure and prey availability) amongst the sampling sites along the study section of the River Bullaque. Moreover, preliminary statistical analyses (e.g. Generalized Linear Mixed Models, GLMMs) with ‘site’ as ‘random’ factor indicated no significant effect. Therefore, data collection was independent across sampling sites and consequently the effect of pseudo-replication was considered non-significant. Finally, the present set of statistical analyses (see below) was chosen as the most appropriate analytical approach, which consisted of pooling all data from the different sampling sites, and this allowed revealing clearer patterns on both aggression and environmental contexts.

Chi-square (χ^2^) tests were performed to determine associations between Aggressor size and: 1) the type of the Aggression; 2) the Shoal size; 3) the Activity prior to the aggressive event; 4) the Recipient species identity; and 5) the Response from the Recipient species elicited by the aggressive display. Specifically, a log-linear analysis was performed to assess associations between Aggressor size, Recipient species, its Response and the interactions of these three factors. However, there were too many cells containing zeroes and thus, two independent chi-square tests were eventually used, in particular for the fourth and the fifth associations (see above).

To test for associations between Aggressor size and the environmental context of the aggressive event, a permutational multivariate analysis of variance (PERMANOVA), with pumpkinseed size class as the fixed factor, was performed on the eight environmental variables, following normalisation and using a Euclidean dissimilarity measure. PERMANOVA was followed by *a posteriori* pair-wise comparisons between size classes. Canonical discriminant analysis of the principal coordinates (CAP) was then used in support to PERMANOVA to visualize the particular patterns of variation between size classes and to select the most influential environmental variables based on a Spearman rank correlation coefficient with the first CAP axis, specifically |ρ| ≥0.3 [38]. Chi-square tests were performed with SPSS v.19 (SYSTAT Software Inc., Chicago, USA) and multivariate analyses were carried out in PERMANOVA+ for PRIMER v.6 [39] with 9999 permutations of the raw data. Significance level for all tests was set at α = 0.05.

## Results

**Figure 1:**
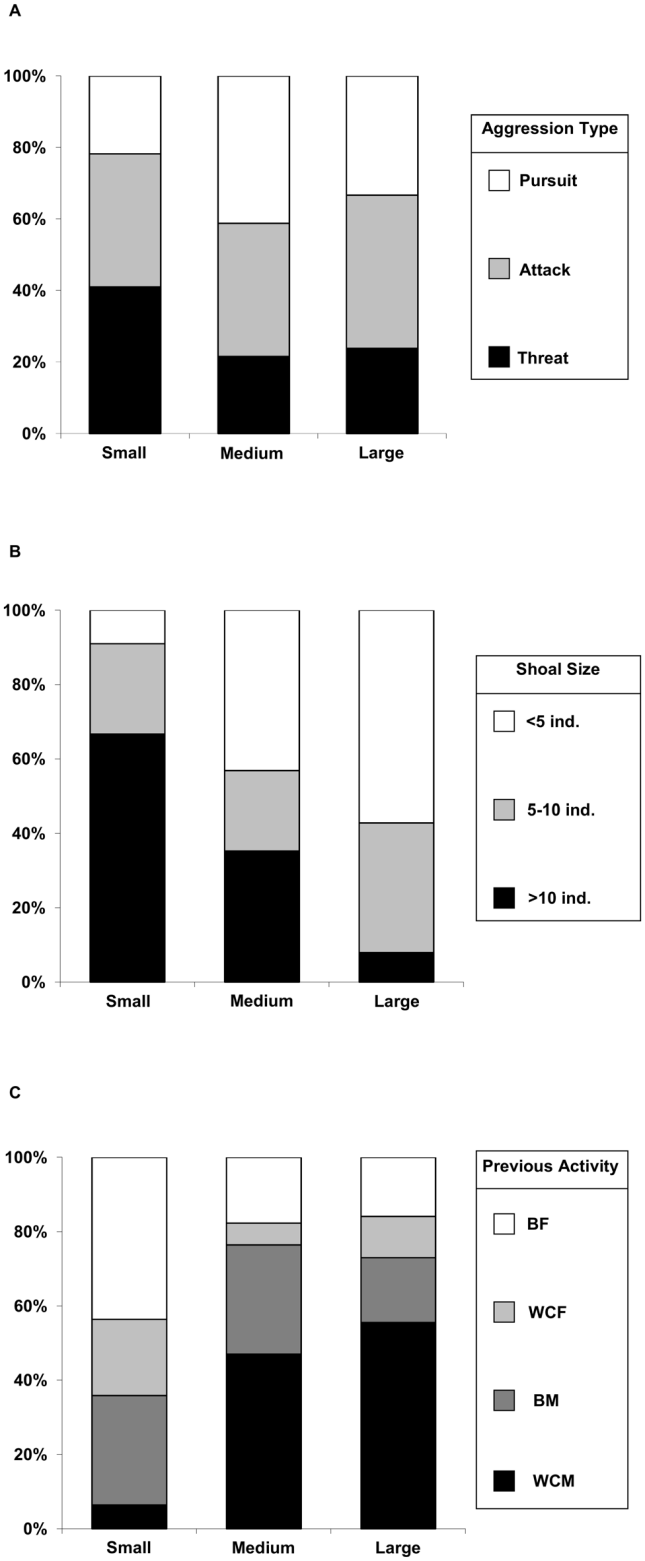
Percentages for three parameters of the aggression context by pumpkinseed size classes: A) Aggression type, B) Shoal size and C) Activity prior to aggression (BF = bottom feeding; WCF = water column feeding; BM = bottom movement; WCM = water column movement).

In total, *n* = 192 aggressive events were recorded: 78 small, 51 medium and 63 large pumpkinseed. Small pumpkinseed showed a higher proportion of Threat and a lower proportion of Pursuit behaviours relative to medium and large individuals (χ^2^ = 9.61, *df* = 4, *p* = 0.048; Figure 1a). The proportion of Aggressors emanating from large shoals (>10 individuals) was higher for small pumpkinseed and decreased with Aggressor size, whereas the proportion of aggressors from small shoals (<5 individuals) was lower and increased with fish size (χ^2^ = 58.89, *df* = 4, *p*<0.001; Figure 1b). Previous Activity to the aggressive event differed between size classes, with small pumpkinseed showing a lower proportion of WCM and a higher proportion of BF relative to medium and large-sized aggressors (χ^2^ = 49.04, *df* = 6, *p*<0.001; Figure 1c). The number of Recipient species subjected to aggression by small, medium and large pumpkinseed was four, eight and seven, respectively, including non-native crayfish and fishes, and more importantly, endemic fishes and frogs (χ^2^ = 45.24, *df* = 14, *p*<0.001; Table 1). The most frequent Recipient species were endemic calandino and non-native eastern mosquitofish. The most frequent Response by the Recipient species following aggression by pumpkinseed of all size classes was RNR, especially in response to medium and large size pumpkinseed, as RR was also a frequent behaviour after aggression from small pumpkinseed (χ^2^ = 37.68, *df* = 6, *p*<0.001; Table 1).

**Table 1:** Percentages for different Responses (NR = no response; AGR = aggression; RR = retreat with return; RNR = retreat with no return) of Recipient species to aggression by pumpkinseed size classes.

	Small				Medium				Large			
Recipient species	NR	AGR	RR	RNR	NR	AGR	RR	RNR	NR	AGR	RR	RNR
*P. clarkii* (54%)	–	4	–	6	2	2	–	8	–	5	–	2
*Luciobarbus* spp. (2%)	–	–	–	–	–	–	–	6	–	–	–	–
*S. alburnoides* (13%)	3	–	12	23	8	–	4	26	5	–	–	23
*S. pyrenaicus* (3%)	–	–	–	–	–	–	–	2	–	2	–	13
*C. paludica* (6%)	3	–	–	9	–	–	–	6	2	–	–	8
*G. holbrooki* (15%)	1	–	25	14	–	–	6	22	3	–	–	23
*M. salmoides* (2%)	–	–	–	–	2	2	2	–	–	–	–	2
*Pelophylax perezi*	–	–	–	–	–	–	–	2	2	–	–	10

Relative abundances are given for every Recipient species of fish and crayfish as the percentage of density in the study area, the remaining 5% is for pumpkinseed (data from [49]).

**Figure 2:**
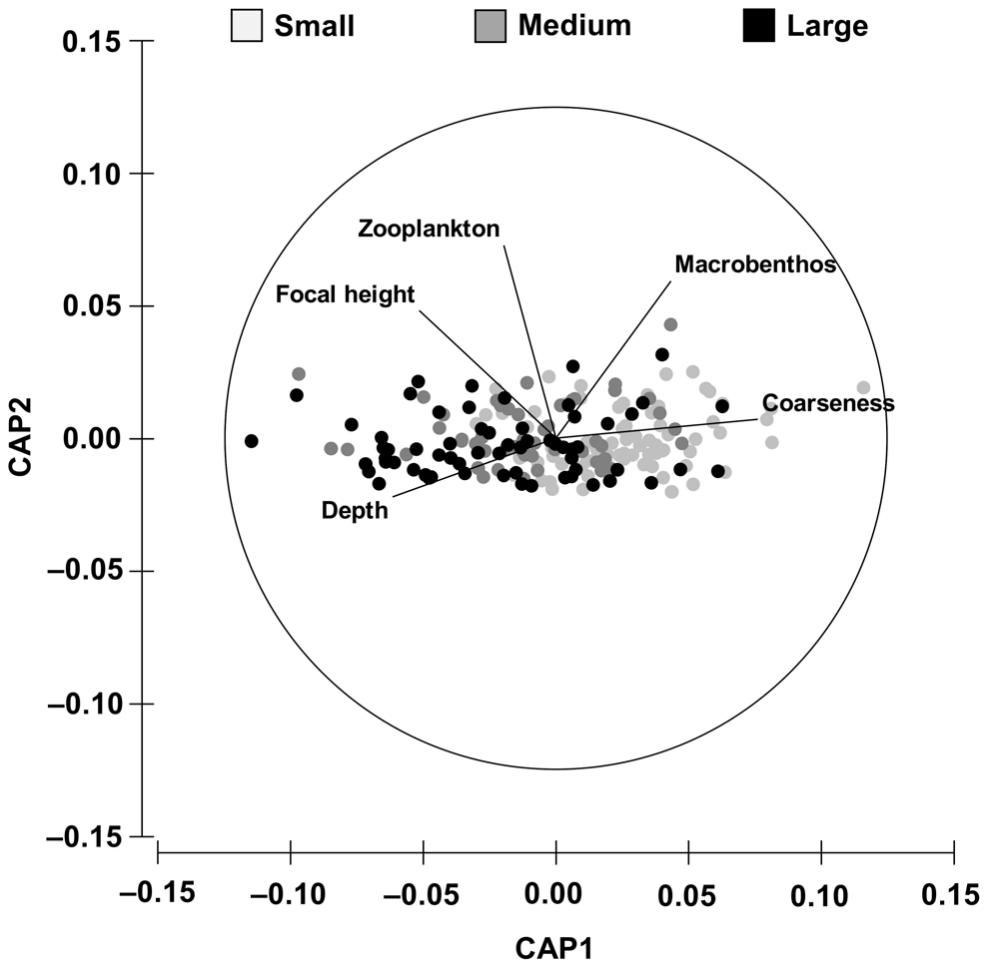
CAP plot for the environmental context of pumpkinseed aggressive behaviour. The selected environmental variables and size classes are shown. See Table 2 for PERMANOVA results.

Environmental context was significantly different between Aggressor size classes, with small pumpkinseed showing clearer differences than medium and large-size pumpkinseed (Table 2). Specifically, aggression by small pumpkinssed was associated with higher Coarseness and lower Depth and Focal height values. Aggression was displayed by medium-sized pumpkinseed where Zooplankton biomass was higher and by large pumpkinseed in points with higher Depth and lower Macrobenthos biomass (Figure 2 and Table 3).

**Table 2:** PERMANOVA on the environmental variables (i.e. microhabitat structure and prey availability) with pair-wise comparisons between pumpkinseed size classes.

Source	df	MS	*F* ^#^/*t*	*p^#^*
Pumpkinseed size class	2	48.28	6.37	<0.001
Small *vs*. Medium			2.47	<0.001
Small *vs*. Large			3.17	<0.001
Medium *vs*. Large			1.54	0.015
Residual	189	7.57		

*F* ^#^ = permutational *F* value; *t* = *t*-test value; *p* ^#^ = permutational *p* value. See Figure 2 for CAP results.

**Table 3:** Environmental variables (i.e. microhabitat structure and prey availability) selected in the CAP by pumpkinseed size classes.

Variable	Small	Medium	Large
Focal height (%)	29.74±3.21	45.20±4.11	49.05±3.78
Water depth (cm)	41.14±1.83	53.24±4.16	70.25±4.60
Coarseness index (1–5)	3.04±0.09	2.53±0.10	2.54±0.10
Zooplankton biomass(mg DM m^–3^)	24.54±2.97	38.27±7.62	26.52±3.69
Macrobenthos biomass(g DM m^–2^)	4.62±0.51	3.99±0.46	2.85±0.43

Results are means ± SE. DM = dry mass.

## Discussion

The degree of complexity and intensity in a fish species’ display of aggressive behaviours can be modulated by the aggressor’s size and stage of development (e.g. [40], [41]). In pumpkinseed, which undergo strong ontogenetic shifts in biological traits [4], [8], [13], the first stage of aggression (i.e. Threat) was observed more frequently in small individuals than in medium and large-sized individuals, which more frequently displayed Pursuit, i.e. the strongest behavioural intensity as final stage of a complete aggressive display [41]. However, medium and large pumpkinseed showed similar frequencies for all aggression types, which indicates that the display of a specific aggressive behaviour is initially size-related and subsequently is employed with a similar frequency from a particular size on. This ontogenetic pattern has also been reported for feeding and reproduction behaviours in fish [42], [43]. These findings provide insight into the potential impact of pumpkinseed on native species, as the most intensive types of aggression are displayed by an abundant component of the pumpkinseed population, the medium and large-sized individuals. For example, the Pursuit type of aggression reached up to 30–40% of all aggressive events in individuals >70 mm TL, i.e. ≈40% of the whole population in the study area [5], [8]. Indeed, the main Response of the Recipient species to aggression from all sizes of pumpkinseed was retreat, specifically with no return (i.e. the strongest behavioural impact of the aggression) when Aggressors were medium or large pumpkinseed. These results highlight the true potential for adverse impact of pumpkinseed through behavioural interference, resulting in the physical displacement of native species from essential resources (e.g. food or habitat), with the subsequent expenditure of energy to avoid the Aggressor (see [26], [33] for other examples in non-native fishes).

In relation to Recipient species, our results showed that pumpkinseed, particularly medium and large sizes, can display aggression on a wide range of taxonomic groups with different ecological requirements, including species at the stream margins (mosquitofish, frog), in the water column (calandino, chub) or on the river bed (crayfish, loach). Previous studies have also shown impacts of pumpkinseed on a variety of functional groups, including zooplankton [19], macrobenthos [44], crayfishes [45], fishes [15] and amphibians [46]. However, this is not always the case. For example, the assumed impact of introduced pumpkinseed on native Eurasian perch [47] has not been supported by recent experimental studies [48]. In the present study area, endemic calandino and non-native eastern mosquitofish were the most frequent recipient species, irrespective of Aggressor size, probably because of their high abundance in the River Bullaque and the overlap of habitat requirements with pumpkinseed [49]. Red-swamp crayfish is also very abundant in this water course [37], [49], and its benthic habits led to numerous encounters with pumpkinseed (D. Almeida, pers. observ.), although these resulted in relatively few aggressive behaviours by pumpkinseed towards red-swamp crayfish. This is presumably because red-swamp crayfish displays high levels of aggression towards fishes (see [50] and present results in Table 1). The eastern mosquitofish has also been shown to display high levels of aggression on the Iberian cyprinid calandino [21]. These three invasive species, i.e. red-swamp crayfish, eastern mosquitofish and pumpkinseed, are the most abundant amongst non-natives in the River Bullaque [49] and consequently, their aggressive behaviours towards endemic species can have profound adverse consequences for aquatic community structure and function. Furthermore, the complexity (i.e. number of species and families) of endemic fish assemblages is low in the Iberian Peninsula [51]. Therefore, fish and also amphibian species may be naive and poorly adapted (i.e. lacking adequate responses) to novel behavioural interactions, especially aggression, and to predation (e.g. [52], [53]), rendering this endemic fauna much more vulnerable to the bioinvasion impacts [22].

Aggression from small pumpkinseed was mainly by Aggressors emanating from large shoals, which afford individuals greater protection and increased foraging rates [54], [55]. Indeed, feeding at the bottom was the most frequently observed activity of small pumpkinseed prior to an aggressive event, particularly over coarse substrata, where macrobenthos biomass was elevated. This was reflected in the low mean Focal height and low Depth, which offer small fish reduced turbulence, and therefore reduced energy expenditure, as well as protection from predators [56] such as non-native largemouth bass in the study area [53]. Conversely, aggressive behaviours by the medium and large-sized pumpkinseed were more common further up in the water column (i.e. Focal heights of 45–50%). This position in the water column can lead to microhabitat overlap with endemic species of barbels, chubs and nases (genus *Pseudochondrostoma*) (see particular focal heights in [36]), which increases the likelihood of aggression by pumpkinseed. The ontogenetic shift in the aggressive behaviour and the habitat use may explain the particular results for medium pumpkinseed, which were observed in shoals and depths of values half-way between small and large pumpkinseed. Particularly regarding prey availability, Zooplankton biomass was more elevated at points where medium pumpkinseed displayed aggression, although this may simply reflect microhabitat partitioning amongst the medium and large-sized pumpkinseed [57], as feeding was not a frequent activity prior to aggression for those size intervals. The results about aggression and environmental contexts suggest that the potential motivation of small pumpkinseed for displaying aggressive behaviours is competition for food, whereas competition for space (e.g. territory defence) is the more likely motivation for aggression by medium and large pumpkinseed.

Although the adverse effects of introduced fishes on native species are usually attributed to predation or exploitative competition, behavioural interference can also make a significant contribution [20] that gives rise to a sinergistic effect, which would otherwise be under-estimated without use of direct observation [21]. In particular, experimental approaches have clearly demonstrated that aggression by non-native fishes affects foraging success [26], [33], [58], reproduction [25] and microhabitat selection [59] of native species. However, field-based studies, such as the present paper, provide more reliable and realistic evidence than do experiments regarding the potential impact of behavioural interference by non-native species [21]. Despite the present study being only able to show potential implications, given that it does not truly measure the negative effects on native species, studies such as presented here should be considered as a first approach to assess the actual impact of aggression. Indeed, this paper provides detailed information with which to design further investigations, both for laboratory experiments and field surveys. Specifically, future research should focus on particular aggressor sizes, aggressive behaviours, recipient species and environmental variables to simplify the assessments and thus better quantify the actual impacts on the fitness of native biota (e.g. abundance, body condition, stress level). Overall, we demonstrate how pumpkinseed can disturb, through aggression, the natural behaviours of endemic fauna in Iberian fresh waters, and it highlights the usefulness of direct *in situ* observations to identify aggressive encounters and quantify these under-estimated impacts of invasive species [21].
